# Deciphering Persistent D‐Dimer Elevation in Paraquat Poisoning: A Mortality Assessment Case Study

**DOI:** 10.1002/ccr3.71677

**Published:** 2025-12-10

**Authors:** Behrooz Hashemi Domeneh, Ali Parouhan, Kimia Mohammadi, Masoumeh Amiri, Sasan Shafiei

**Affiliations:** ^1^ Department of Medical Science Tehran Pezeshki Branch, Islamic Azad University Tehran Iran; ^2^ Students Research Committee Iran University of Medical Sciences Tehran Iran; ^3^ Student Research Committee Tehran Pezeshki Branch, Islamic Azad University Tehran Iran; ^4^ Faculty of Medicine Kermanshah University of Medical Science Kermanshah Iran; ^5^ Department of Cardiology, School of Medicine Shiraz University of Medical Sciences Shiraz Iran

**Keywords:** coagulation, D‐dimer, oxidative stress, paraquat intoxication, pulmonary embolism

## Abstract

A 74‐year‐old woman accidentally ingested an unknown amount of paraquat without suicidal intent. Quickly presenting with dysphagia and tongue and oral mucosa burns, she sought medical care. Admitted to the intensive care unit, by the third day of hospitalization, due to the need to be evaluated for respiratory problems. A surge in the patient's blood D‐dimer level prompted concerns of possible pulmonary embolism; however, subsequent evaluations ruled out pulmonary embolism or other complications. This case reinforces the hypothesis that the elevation in the blood's D‐dimer level might be associated with paraquat poisoning itself.

## Key Clinical Message

In paraquat poisoning, persistent D‐dimer elevation can occur without evidence of thromboembolism. This finding indicates that paraquat's toxicity may extend to coagulation, necessitating careful monitoring of D‐dimer levels regardless of typical complications.

## 1 Introduction

Paraquat, also known as PQ or 1,1′‐dimethyl‐4,4′‐bipyridinium dichloride, is a rapid‐acting, nonselective herbicide extensively employed in the Asia‐Pacific area, notably in countries like China, Korea, and Sri Lanka [1, 2, 3]. Because of its widespread availability, incidents of PQ poisoning are frequent. Both deliberate and accidental ingestion of PQ pose a significant public health challenge, particularly in developing nations [4]. While dermal contact or exposure through spraying typically causes localized and limited injury, accidental or deliberate ingestion leads to an alarmingly high fatality rate. Beyond severe local irritation in the mouth, oropharynx, and esophagus, it can result in multiple organ failure involving the heart, lungs, liver, and kidneys. Pulmonary symptoms typically take precedence and frequently result in fatalities.

Two hours post oral ingestion, the highest concentration of PQ in plasma is observed, and within the initial 24 h, most of the originally ingested PQ is eliminated through renal excretion. The production of reactive oxygen species within cells, leading to cellular harm via lipid peroxidation, activation of nuclear factor kappa B, mitochondrial damage, and apoptosis, is the primary cause behind the clinical manifestations of PQ poisoning. Despite concentration gradients, PQ easily infiltrates lung tissue, inducing lung fibrosis and pneumonitis [4, 5].

Coagulation is the process that transforms blood from a liquid to a gelatinous state, vital for hemostasis. It involves platelets, the vascular endothelium, and a sequence of reactions resulting in fibrin formation after endothelial injury. While well understood in general human coagulation, pivotal indices used in clinical practice include PT, INR, Fbg, APTT, and TT. However, the specific mechanisms of coagulation involved in PQ poisoning remain unclear [6, 7].

Herein, we discuss a case of a 74‐year‐old female with a highly suggestive history of incidental PQ ingestion who presented with complaints of oral ulcers, dysphagia, and drooling.

## 2 Case History/Examination

**FIGURE 1:**
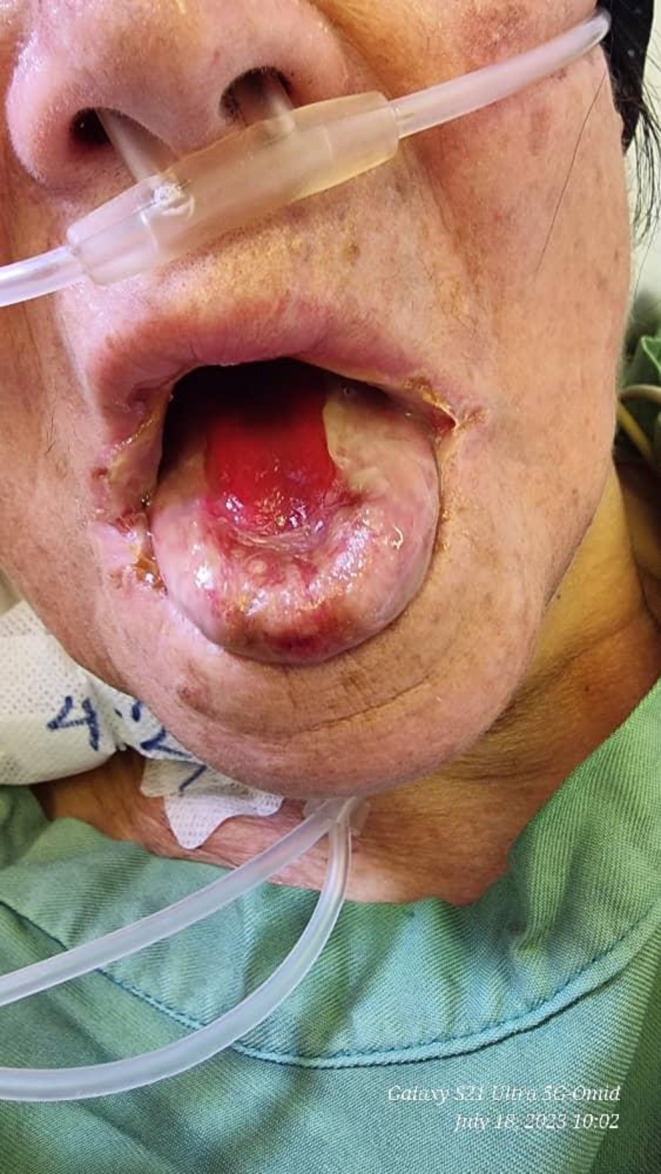
Close‐up view of oral lesions in the patient, demonstrating severe burns and ulcerations on the labial mucosa following paraquat ingestion.

A 74‐year‐old female was brought to the emergency department of our academic hospital with an alleged history of PQ poisoning, according to the photo of the bottle of the herbicide, ingestion 2 days ago, followed by multiple oral ulcerations with erythematous borders, tongue pain, right side sialorrhea, and difficulty swallowing for 2 days (Figure 1).

The patient was clinically diagnosed with PQ poisoning, based on the characteristic symptoms associated with poisoning in the oral cavity and the information provided by the patient and their relatives upon admission, including the photograph of the herbicide bottle.

There was no history of vomiting, loose stools, abdominal pain, seizures, or fever. She had a past medical history of right‐sided hearing impairment for 10 years. She was admitted to a local hospital after reporting that she had accidentally ingested 1–2 mouthfuls of PQ (50 cc), mistaking it for a soda beverage. Gastric lavage and one dose of atropine were administered. She was referred to our hospital 2 days later and was admitted to the intensive care unit. During the examination, she remained conscious, attentive, and oriented. Her Glasgow Coma Scale (GCS) score was 15/15.

Multiple oral ulcers were detected intraorally, distributed across the tongue, gingiva, labial mucosa, and buccal mucosa, presenting in diverse sizes. Her vital signs showed a pulse rate of 98 beats/min, regular blood pressure of 130/80 mm Hg, and a respiratory rate of 22 breaths/min, with an SpO_2_ of 98% on ambient air. Normal heart sounds were detected, and chest auscultation revealed clear breath sounds in both lungs and no heart murmur. Her pupils were equal in size and responded to light. Dexamethasone was administered at admission. In addition, chlorhexidine mouthwash was used every 8 h. Her initial chest radiograph showed no infiltrates and was normal.

## 3 Differential Diagnosis, Investigations, and Treatment

A non‐contrast CT scan of the chest and mediastinum was performed with the result of bilaterally scattered band atelectasis and a well‐defined solid nodule measuring 7 × 6 mm with a subpleural location and angular border seen in RML. Also, an age‐related subpleural fibro cyst was seen in the dependent area. Gastrointestinal tract protection by intravenous (IV) drip of 80 mg pantoprazole sodium was done. Her baseline investigations revealed microcytic anemia suggestive of iron deficiency (RBC:3.73, HGB: 9.5 g/dL, M.C.V: 78.6 fl), elevated ESR (48 mm/h) and CRP (32.9 mg/dL), raised bilirubin (total: 2.4 mg/dL, direct: 0.59 mg/dL, indirect: 1.8 mg/dL), and low levels of calcium (7.5 mg/dL) and magnesium (1.4 mg/dL). Other biochemistry markers, hematological parameters, cardiac enzyme levels, and electrolytes were within the normal range. Shaldon catheterization was conducted during the initial hours, followed by a 4‐h hemoperfusion for the patient.

During hospitalization, the patient was bedridden and NPO. On the sixth day of admission, baseline laboratory tests showed normal cardiac troponin 1, CK‐MB MASS, PT, PTT, INR, and other lab data, but CK‐MB‐total (5.16 ratio) and CPK (197 U/L) were elevated; therefore, D‐dimer was checked (it was above 5000 ng/mL). Given this significant elevation, a comprehensive workup was undertaken to exclude other potential causes beyond thromboembolism. Disseminated intravascular coagulation (DIC) was ruled out based on the normal platelet count, PT, PTT, and INR. Underlying malignancy was considered unlikely in the absence of clinical signs, weight loss, or suggestive findings on thoracic CT imaging. Significant hepatic dysfunction was excluded based on near‐normal liver enzyme levels (AST, ALT), and the patient had no signs of active infection or systemic inflammatory conditions aside from the localized chemical injury from paraquat. The patient revealed no symptoms of chest pain, chest tightness, or shortness of breath. On physical examination, the breath sounds in the lungs were clear, with no rhonchi or rales.

Electrocardiography displayed no signs of arrhythmia or cardiac abnormalities. Echocardiography was performed, and a normal result with an ejection fraction of 55% was reported. Also, lower extremity venous color Doppler ultrasonography showed no evidence of thrombosis or DVT on both sides. Furthermore, CT angiography findings indicated the absence of pulmonary embolism in the major branches of the right and left pulmonary arteries, as well as in the segmental branches. Moreover, degenerative joint disease changes and a reduction in the lordosis of the thoracic vertebrae were observed.

After requisite diagnostic interventions, a re‐assessment of the patient's D‐dimer levels was conducted (2217 ng/mL). Other hematological markers, such as PT, PTT, INR, etc. were within the normal range.

## 4 Conclusion and Results (Outcome and Follow‐Up)

The patient was discharged without any prophylactic treatment for DVT and PTE.

The elevation of D‐dimer levels in a PQ poisoning case, devoid of expected thromboembolism or other medical problems, introduces a novel aspect in understanding this toxicity. Investigating this D‐dimer increase could unveil critical insights into paraquat's broader influence on coagulation, offering new dimensions in managing and understanding its toxicity.

## 5 Discussion

The case presented here illustrates a 74‐year‐old female with a clinically diagnosed history of PQ ingestion. The initial symptoms primarily involved multiple oral ulcerations, tongue pain, sialorrhea, and dysphagia. Despite an early diagnosis and appropriate management, the patient exhibited persistent abnormalities in D‐dimer levels, indicating ongoing coagulation activation despite the absence of apparent cardiac or pulmonary complications.

Paraquat (1,1′‐dimethyl‐4,4′‐bipyridinium dichloride) intoxication is notoriously characterized by a high mortality rate, primarily due to progressive multiple organ failure [1, 4]. Its toxicity is largely mediated through cyclic redox reactions, generating an overwhelming surge of reactive oxygen species (ROS) such as superoxide anions (O_2_•^−^) and hydroxyl radicals (•OH) [1]. This oxidative stress induces catastrophic cellular damage via lipid peroxidation, mitochondrial dysfunction, and apoptosis. The lungs are the primary target organ due to the selective uptake of paraquat by alveolar epithelial cells, leading to diffuse alveolar damage that progresses from an initial destructive phase to irreversible pulmonary fibrosis and respiratory failure [1, 4, 6]. However, the systemic nature of the toxicity also frequently results in acute kidney injury, hepatic necrosis, myocardial damage, and neurological complications, creating a profound systemic inflammatory response syndrome that often culminates in death [1, 4, 6, 8].

The persistence of elevated D‐dimer levels in the absence of evident cardiac or pulmonary complications raises intriguing clinical considerations. While PQ poisoning primarily manifests with pulmonary and multi‐organ complications, the ongoing elevation of D‐dimer, an indicator of fibrin degradation, might suggest an underlying pro‐coagulant state or an ongoing systemic inflammatory response. This finding, notably inconsistent with the absence of observable thromboembolic events, underscores the complexity of PQ toxicity and its potential systemic impact beyond traditional organ‐specific manifestations.

The precise molecular mechanism linking paraquat poisoning directly to D‐dimer elevation remains to be fully elucidated [1]. However, a plausible pathway can be hypothesized based on its established toxicity. Paraquat's primary mechanism of action is the cyclic generation of reactive oxygen species (ROS), leading to profound oxidative stress. This oxidative insult is known to cause direct damage to vascular endothelial cells [1, 8]. Endothelial injury is a well‐known potent trigger of the coagulation cascade [9]. It promotes the release of tissue factor, activates platelets, and downregulates natural anticoagulant mechanisms, leading to increased thrombin generation and fibrin formation [9, 10]. While this fibrin formation may not organize into clinically apparent thromboemboli, it is likely undergoing constant, low‐grade breakdown by the fibrinolytic system [10, 11, 12]. This process of heightened fibrin formation and degradation would be directly reflected by an elevation in D‐dimer, a specific degradation product of cross‐linked fibrin. Therefore, the observed D‐dimer elevation in our patient may represent a state of subclinical, disseminated intravascular coagulation (DIC) or localized intravascular coagulation driven by paraquat‐induced endothelial damage and systemic inflammation, without progressing to full‐blown thrombosis.

The observations align with existing knowledge regarding PQ‐induced oxidative damage and multi‐organ toxicity but introduce a novel aspect concerning coagulation dynamics. Limited literature exists on persistent D‐dimer elevation in PQ poisoning without concurrent thrombotic events, thus highlighting a potential avenue for further investigation into paraquat's systemic effects on coagulation pathways.

Previous studies have frequently highlighted PQ‐induced toxicity, attributing it to the production of free radicals and oxidative stress. Metabolism of PQ through various enzyme systems generates an abundance of superoxide (O_2_^−^), triggering the formation of hydroxyl free radicals (HOs), causing oxidative harm and apoptosis. The plasma concentration of PQ peaks within 2–4 h post oral ingestion and often remains relatively stable for days. Due to the lung's significant vascularity, PQ accumulates actively in lung tissues during this period. Hence, the lungs typically suffer the most severe damage in PQ poisoning cases [4, 13, 14, 15, 16, 17].

While it is established that acute PQ poisoning can disrupt blood coagulation in humans, uncertainties persist regarding the potential relationship between the amount ingested and the severity of coagulation dysfunction. Furthermore, limited research has explored how coagulation status might influence the prognosis of individuals affected by PQ poisoning [8].

Kubo et al.'s research [18] indicated a link between plasma D‐dimer levels and mortality in individuals experiencing the acute progression of idiopathic fibrosis. Employing anticoagulant therapy might enhance patient survival. Correspondingly, animal models were created by Liu Feng et al. [19]. However, Xiao Hu et al. [8] did not discern a clear correlation between alterations in D‐dimer levels and patient survival.

However, Su‐Jin Seok et al. [20] investigated D‐dimer levels among patients experiencing acute PQ intoxication, revealing reduced D‐dimer levels in the PQ group when compared to the control group. Although the PQ group exhibited higher tissue plasminogen activator (tPA) and plasminogen activator inhibitor‐1 (PAI‐1) levels, the lower D‐dimer levels suggested a possible decline in fibrinolytic activity in PQ intoxication. These findings propose that paraquat‐related reactive oxygen species (ROS) could influence fibrinolytic function by suppressing D‐dimer levels.

In summary, PQ ingestion can lead to considerable damage to vascular endothelial cells, causing coagulation issues upon entering the blood [21].

Similarly, femoral vein catheterization, a standard medical procedure, may induce endothelial injury and subsequent clot formation, potentially resulting in pulmonary thromboembolism. Moreover, the insertion of a double‐lumen catheter through a central vein during hemoperfusion can also prompt pulmonary thromboembolism [22].

Taken together, if a patient treated for PQ poisoning demonstrates an isolated increase solely in D‐dimer levels while other diagnostic tests and procedures show normal results, it is likely attributed to PQ intoxication. This scenario suggests that cardiac or pulmonary issues are less probable and highlights the influence of PQ poisoning on the patient's condition.

One of the limitations of this study is the lack of measurement of the concentration of PQ in the patients' blood. The initiation of treatment was based on the reported oral PQ intake history and the presence of the poison container held by the patients' family.

## Author Contributions

**Behrooz Hashemi Domeneh:** conceptualization, project administration, supervision, validation, visualization. **Ali Parouhan:** writing – original draft, writing – review and editing. **Kimia Mohammadi:** data curation, investigation. **Masoumeh Amiri:** writing – original draft, writing – review and editing. **Sasan Shafiei:** methodology.

## Funding

The authors have nothing to report.

## Ethics Statement

For a case report, ethical approval is waived at our institution.

## Consent

Written informed consent was obtained from the patient's legal representative for publication of this case report and any accompanying images. The complete consent form is included as an appendix to this manuscript.

## Conflicts of Interest

The authors declare no conflicts of interest.

## Data Availability

The data that support the findings of this study are available on request from the corresponding author. The data are not publicly available due to privacy or ethical restrictions.

## Case Report—Patient Consent Form

Name:

MRN:

DOB:

The purpose of a case report is to share new, unique information experienced by one patient during his/her clinical care that may be useful for other physicians and members of a health care team. A case report may be published in print and/or via internet dissemination for others to read, and/or presented at a conference.

**I understand that:**

1. We are obligated to protect your privacy and not disclose your personal information (information about you and your health that identifies you as an individual, e.g., name, date of birth, medical record number). When the case report is published or presented, your identity will not be disclosed.
2. Although your personal information collected or obtained will be kept confidential and protected to the fullest extent of the law, there is a limited risk associated with this case report that could result in a loss of confidentiality due to your unique experience.
3. Taking part in this case report is voluntary. You may choose not to take part, or you may change your mind at any time. However, once the case report is written and published, it will not be possible for you to withdraw from it. Your decision will not result in any penalty or loss of benefits to which you are entitled, including the quality of care you receive.
4. I will not receive any financial benefits from the publication of this case, and allowing your information to be used in this case report will not involve any additional costs to you.
5. The physician has fully explained to me the nature and purpose of a case report, the options, and the possibility of withdrawal.
6. I certify that I have read and fully understand the information presented in this consent. In addition, I have been afforded an opportunity to ask whatever questions I have regarding the case report. My questions have been answered to my satisfaction.

**Comments:**

*Patient/Authorized Representative (relationship)       Date     Time*.

*Witness Signature                    Date     Time*.

*Physician Signature          Print Name       Date    Time*.

**Principal Investigator:**__________________________________________________________.

**Department:** ___________________________________  **Phone:**_______________________________.
